# Analysis on the related factors of the influence of high-quality nursing service in cardiology department on patients’ psychological mood and rehabilitation index

**DOI:** 10.1097/MD.0000000000043706

**Published:** 2025-08-08

**Authors:** Jialin Wang, Zhong Ye, Qin Xie

**Affiliations:** a Department of Cardiology, West China Hospital of Sichuan University, Chengdu City, Sichuan Province, China; b School of Basic Medical Sciences, Chengdu Railway Health School, Chengdu City, Sichuan Province, China.

**Keywords:** complications, coronary heart disease, quality nursing service, satisfaction

## Abstract

In the field of cardiology, high-quality nursing service has been recognized as an important factor to improve the health effect of patients. However, there is still a lack of research on how these services specifically affect patients’ psycho-emotional and recovery indicators. Therefore, analyzing the effects of cardiology quality nursing services on patients’ psychological emotions and recovery indicators and their correlation factors is of great significance for improving the quality of nursing care and patients’ overall recovery effects. The objective of this study is to investigate the positive effect of quality nursing services on the rehabilitation process of coronary artery disease patients hospitalized. In this study, a retrospective study was conducted to collect clinical data related to patients with confirmed coronary artery disease who attended the cardiology department of our hospital during the period of June 2020 to December 2023 and were treated with percutaneous coronary intervention. A total of 150 patients were eligible for enrollment, including 85 males and 65 females. The patients were randomized to the experimental group and the control group to receive quality nursing service and routine nursing service, respectively. The hospitalization time, complications, and satisfaction with diagnosis and treatment activities of the 2 groups were collected, and the scientificity and effectiveness of quality nursing service were analyzed. The analysis showed that the hospitalization time in the experimental group (6.83 ± 0.45) days was shorter compared to that of the control group (7.16 ± 0.55) days and the difference was statistically significant (*P* < .05). Patients in the experimental group had lower scores on the anxiety self-assessment scale and depression self-assessment scale than those in the control group, and the difference was statistically significant. The complication rate of the experimental group was significantly lower than that of the control group, and the patients in the experimental group were more satisfied with the treatment process during their hospitalization than those in the control group. Quality nursing service is a more effective way of inpatient care, compared with traditional care and can be more effective in reducing the length of stay, reduce the incidence of complications, and improve the patient’s satisfaction with the nursing staff.

## 1. Introduction

As one of the major global health problems, cardiovascular diseases have a serious impact on the quality of life and mental health of patients.^[1,2]^ With the continuous progress of medical technology and the continuous updating of nursing concepts, quality nursing service in cardiology has become an important means to improve the treatment effect and the quality of rehabilitation of patients. In recent years, studies at home and abroad have shown that quality nursing services can not only improve the physiological condition of patients with cardiovascular diseases, but also significantly enhance their psychoemotional state, thereby promoting the recovery process.^[3,4]^ A study by Reiter-Brennan et al found that comprehensive nursing interventions (including psychological support, health education, and personalized care plans) significantly improved patients’ emotional state and contributed to faster recovery. In addition, multidimensional assessments and interventions in nursing services have been shown to positively contribute to patients’ mood and recovery indicators.^[5]^ In China, as the concept of quality nursing services has become more widely understood, the number of relevant research studies has increased. The current study highlights the positive role of patient-centered care models in improving psychological status and rehabilitation outcomes for patients with cardiovascular disease.^[6–8]^ Particularly in the Chinese context, studies have shown that personalized psychological interventions and health education have a significant effect on relieving patients’ psychological stress and improving recovery indicators. However, domestic research on the specific impact of quality nursing services on cardiology patients’ psychoemotional and rehabilitation indicators is still relatively limited, and systematic analyses of correlating factors are still insufficient. In summary, although existing studies have revealed the important impact of quality nursing services on patients with cardiovascular diseases, there is still a need to further explore their specific mechanisms of action and correlates in different contexts. The aim of this study was to reveal the potential correlations by systematically analyzing the effects of cardiology quality nursing services on patients’ psychological emotions and recovery indicators, with a view to providing a scientific basis for optimizing nursing intervention strategies.

## 2. Materials and methods

### 2.1. Research objects

In this study, a retrospective study was conducted to collect clinical data related to patients with confirmed coronary artery disease who attended the cardiology department of our hospital during the period of June 2020 to December 2023 and were treated with percutaneous coronary intervention. A total of 150 patients were enrolled, including 85 males and 65 females. We obtained a waiver of informed consent from the Ethics Committee. The Ethics Committee of West China Hospital of Sichuan University approved the study (Ethics approval number: W-2023-1315-02). This study adhered to the Declaration of Helsinki.

### 2.2. Research methods

A total of 150 patients were collected in this study. The patients were divided into 2 groups according to randomization, in which 75 cases in the control group received routine nursing services and 75 cases in the experimental group received quality nursing services. The correlates of length of stay, complications, patient-related emotional data, and satisfaction with healthcare services were collected and statistically analyzed between the 2 groups.

### 2.3. Collection indicators

This study collected patients’ gender, age, body mass index (BMI), hospitalization time, complications, self-rating anxiety scale (SAS),^[9]^ and self-rating depression scale (SDS)^[10]^ reflecting patients’ emotions, and patients’ satisfaction with medical services.

### 2.4. Interpretation of some of the validation indicators mentioned in this study

#### 2.4.1. Self-rating anxiety scale

We used the SAS in order to assess the occurrence of postoperative anxiety in our patients.

#### 2.4.2. Self-rating depression scale

We used the SDS to assess the occurrence of postoperative depression in our patients.

### 2.5. Groups

#### 2.5.1. Control group

A routine nursing care model was adopted, including patiently introducing knowledge about coronary heart disease to patients and their families, monitoring patients’ vital signs, and providing guidance on diet and medication as prescribed by the doctor.

#### 2.5.2. Experimental group

*Health education*: Combined with the patient’s disease cognitive situation and comprehension ability, we reasonably choose the health promotion method, and briefly introduce the treatment method, pathogenesis, and clinical manifestations to the patients.

*Psychological counseling*: Understand the patient’s emotional changes and sources of psychological stress, understand the worries and concerns, etc according to the cause of the patient’s abnormal emotions to carry out appropriate counseling. Through the introduction of successful cases of treatment, patients’ self-confidence in treatment is enhanced, and the importance of maintaining a good state of mind in controlling the progression of the disease is explained.

*Dietary guidance and medication guidance*: Understand the patient’s daily dietary preferences and habits, and then formulate a dietary programme that meets the patient’s needs according to the changes in his or her condition. Patiently and meticulously explain drug-related knowledge, including drug dosage, characteristics, possible adverse reactions, treatment, etc informing of the important role of medication compliance, as well as providing guidance on the daily use of medication, especially drug dosage. Private adjustments of drug dosages are prohibited. Tell patients to carry nitrates with them, and apply them immediately once the illness breaks out to prevent the illness from getting worse.

*Electrocardiogram monitoring and nursing*: Most of the patients were taken for electrocardiogram monitoring immediately after admission, so as to find out whether the patients suffered from cardiac arrhythmia, and to take intervention countermeasures as soon as the abnormality was detected. The condition of the electrode pads and surrounding skin tissue is also monitored to prevent the appearance of redness, swelling, and allergies.

*Angina pectoris intervention*: After the onset of the disease, focus on daily rest, mainly in bed, while monitoring the severity of the pain, understand the change of heart rate, etc. As soon as the number of angina occurrences and worsening of symptoms are detected, feedback is given to the physician and help is given to target the management. Enhance the prevention of myocardial infarction by early detection of precursor symptoms, such as rapid pulse rate and rapid drop in blood pressure, so that early treatment can be taken.

### 2.6. Statistical methods

The data were statistically analyzed using SPSS 25.0 (IBM, Armonk) statistical software. Measurement data is represented by average number ± standard deviation; categorical variables are expressed as counts (percentages). When the measurement data obey the normal distribution, the paired *t* test is used for analysis. When the measurement data does not obey the normal distribution, it is represented by median and interquartile interval, and the rank sum test is used. Descriptive statistical analysis and 1-way analysis of variance were used to analyze the related indexes of patients receiving quality nursing services. Chi-square test was used to verify the value of high-quality nursing service in shortening hospitalization time, reducing complications, and improving medical service satisfaction. *P* < .05 is statistically significant.

## 3. Results

### 3.1. Comparison of baseline characteristics between 2 groups of patients

Patients in both groups were randomly divided into 2 groups: 44 males and 31 females in the experimental group; there were 41 males and 34 females in the control group, *P* = .621 > .05. There was no significant difference between the 2 groups. Comparing the age and BMI of the 2 groups, the *P* value was >.05. There is no statistical difference in age and BMI between the 2 groups (*P* > .05), which means that the 2 groups are balanced and comparable in baseline data level. See Table 1.

**Table 1:** Comparison of baseline characteristics, hospitalization time, and emotional indexes.

Variables	Total (n = 150)	Experimental group (n = 75)	Control group (n = 75)	Statistic	*P*
Age, mean ± SD	64.52 ± 5.36	64.67 ± 5.15	64.37 ± 5.59	*t* = 0.33	.739
BMI, mean ± SD	26.03 ± 1.01	26.01 ± 1.06	26.06 ± 0.96	*t* = −0.33	.742
Gender (male: 1, female: 0), n (%)				*χ*² = 0.24	.621
Male	85 (56.67)	44 (58.67)	41 (54.67)		
Female	65 (43.33)	31 (41.33)	34 (45.33)		
Hospitalization time	6.99 ± 0.52	6.83 ± 0.45	7.16 ± 0.55	*t* = −4.09	<.001
SAS	54.09 ± 7.61	49.92 ± 6.93	58.27 ± 5.76	*t* = −8.02	<.001
SDS	56.49 ± 8.06	49.71 ± 4.99	63.28 ± 3.56	*t* = −19.19	<.001

BMI = body mass index, SAS = self-rating anxiety scale, SD = standard deviation, SDS = self-rating depression scale.

### 3.2. Comparison of hospitalization time and emotional indexes between the 2 groups

In this study, the changes of emotional indexes of patients during hospitalization were evaluated by SAS and SDS. Compared with the control group (7.16 ± 0.55), the hospital stay of patients in the experimental group (6.83 ± 0.45) was shorter, and the difference was statistically significant (*P* < .05). The scores of SAS and SDS in the experimental group were lower than those in the control group, and the difference was statistically significant (*P* < .05). See Table 1.

### 3.3. Incidence of complications of patients

By consulting the patient’s medical records, we can get the complications during hospitalization, including 10 cases of arrhythmia and 9 cases of heart failure in the control group, totaling 19 cases. There were 3 cases of arrhythmia and 2 cases of heart failure in the experimental group, totaling 5 cases. Comparing all the complications between the 2 groups, the incidence of complications in the experimental group was significantly lower than that in the control group, and the difference was statistically significant (*P* < .05). See Table 2.

**Table 2:** Incidence of complications of patients, evaluation results of nursing ability, and patient satisfaction.

Variables	Total (n = 150)	Control group (n = 75)	Experimental group (n = 75)	Statistic	*P*
Arrhythmia	13 (8.7)	10 (13.33)	3 (4.00)	*χ*² = 9.72	.002
Heart failure	11 (7.3)	9 (12.00)	2 (2.67)		
Evaluation results of nursing ability	78.93 ± 6.60	74.61 ± 4.43	83.24 ± 5.51	*t* = −10.57	<.001
Patient satisfaction, n (%)				*χ*² = 4.13	.042
Satisfied	137 (91.33)	65 (86.67)	72 (96.00)		
Unsatisfied	13 (8.67)	10 (13.33)	3 (4.00)		

### 3.4. Evaluation results of nursing ability

The nursing staff in the experimental group and the control group all carried out the corresponding nursing ability assessment. The results showed that the scores of nurses in the experimental group (83.24 ± 5.51) were significantly higher than those in the control group (74.61 ± 4.43), with statistical significance (*P* < .05). See Table 2.

### 3.5. Patient satisfaction survey

Before leaving the hospital, we investigated the patients and asked them about their satisfaction with the hospital’s diagnosis and treatment activities. It was found that the satisfaction of patients in the experimental group was significantly higher than that in the control group, and the difference was statistically significant (*P* < .05). See Table 2.

## 4. Discussion

Cardiovascular disease is a major health problem in the world with its high incidence. And disability rate make comprehensive management of such diseases particularly important.^[11,12]^ Epidemiological studies show that the occurrence and development of cardiovascular diseases are not only closely related to biological factors. However it is also influenced by psychological factors, lifestyle, and social support. Patients with cardiovascular diseases are often accompanied by high psychological stress and negative emotions, such as anxiety and depression. These psychological problems not only have a negative impact on the quality of life of patients, but also may have an adverse effect on the prognosis of their diseases.^[13,14]^ The intervention of high-quality nursing service in cardiology, especially in psychological support and personalized nursing, has been gradually incorporated into the management strategy of cardiovascular diseases. Therefore, the implementation of high-quality nursing service is considered to be an important means to alleviate these psychological troubles and improve the overall health of patients. This study systematically analyzes the influence of high-quality nursing service in cardiology department on patients’ psychological mood and rehabilitation index. The results confirmed that the high-quality nursing service in the Department of Cardiology has a significant effect in improving the psychological mood and rehabilitation index of patients with coronary heart disease. This result not only verifies the scientificity of high-quality nursing service, but also provides a strong basis for further optimizing nursing intervention strategies.

### 4.1. Effect of quality nursing service in cardiology department

The results of this study show that high-quality nursing service has a significant impact on the psychological mood and rehabilitation index of patients with coronary heart disease. The patients in the experimental group received high-quality nursing service, which significantly improved the hospitalization time, reduced the occurrence of complications, and improved the satisfaction with the diagnosis and treatment activities. This is consistent with the relevant research results at home and abroad, and supports the effectiveness of high-quality nursing service in cardiovascular disease management. Foreign research shows that comprehensive nursing intervention can significantly improve the psychological state and quality of life of patients with cardiovascular diseases. In particular, personalized nursing, health education, and communication skills for psychological support have been proved to be effective in reducing patients’ anxiety and depression, thus promoting their overall recovery.^[15,16]^ These findings are consistent with our findings, which further supports the role of quality nursing service in psychological intervention and rehabilitation promotion.

### 4.2. Improvement of psychological mood

High-quality nursing service helps to relieve patients’ anxiety and depression by establishing good doctor-patient relationship, providing personalized psychological support and effective health education. The results of this study show that the psychological mood of patients in the experimental group has been significantly improved during hospitalization. It is closely related to the common psychological problems of patients with cardiovascular diseases. Epidemiological data show that patients with cardiovascular diseases often experience a high level of psychological stress due to the seriousness of the disease itself and the pressure of long-term treatment.^[17–19]^ Psychological intervention measures in high-quality nursing service, such as regular psychological evaluation, professional psychological consultation, and emotional support, can effectively reduce the anxiety and depression level of patients. This result is consistent with the research at home and abroad, which shows that psychological intervention has a positive effect on improving the psychological state of patients with cardiovascular diseases.^[19]^

### 4.3. Improvement of rehabilitation index

High-quality nursing service not only has a positive impact on the psychological level, but also has a significant effect on patients’ rehabilitation indicators. The results show that the hospitalization time of patients in the experimental group is significantly shortened, and the incidence of complications is low. High-quality nursing service can improve the rehabilitation effect of patients through comprehensive nursing intervention measures. For example, optimizing the nursing process, strengthening patient health management, and preventing complications in a timely manner.^[20–22]^ High-quality nursing service in cardiology usually includes comprehensive evaluation of patients, individualized nursing plan, and multidisciplinary teamwork. This comprehensive nursing intervention can effectively manage the cardiovascular symptoms of patients, optimize the treatment plan, and improve the rehabilitation effect. Individualized nursing plan can adjust treatment and nursing measures according to patients’ specific conditions. Therefore, it can improve rehabilitation indicators more effectively, such as cardiac function, exercise endurance, and quality of life. In addition, high-quality nursing service pays attention to making personalized nursing plan according to the individual differences of patients. By knowing the patient’s medical history, living habits, and psychological state in detail, the nursing team can design the most suitable nursing plan for each patient. This kind of individualized nursing can better meet the specific needs of patients, improve the effectiveness of treatment, and thus improve the rehabilitation index.

### 4.4. Limitations

This study provides valuable insights on the influence of quality nursing service in cardiology department on patients’ psychological mood and rehabilitation index. However, there are some limitations. First of all, sample selection bias may affect the universality of the results. It is suggested that future research should adopt multicenter and large sample random sampling to enhance the representativeness of the results. Secondly, the sample size may not be enough to comprehensively evaluate the nursing effect. So it is suggested that the sample size should be increased in future research to improve the accuracy of statistical analysis. In addition, psychological and emotional evaluation depends on patients’ self-report, which has subjective bias. Future research should consider combining various evaluation tools to improve the accuracy of the results. The standardization of nursing intervention may also affect the consistency of results. So it is suggested to formulate clear nursing intervention standards. Short-term follow-up time limits the evaluation of long-term effect, and future research should consider longer follow-up. In addition, the influence of individual differences on psychological and emotional intervention may not be fully explored. And future research should pay attention to the effect of personalized nursing. Finally, the change of the epidemiological background of cardiovascular diseases may also affect the applicability of the conclusions. So it is suggested to conduct research at different times and regions to obtain broader conclusions.

## 5. Conclusion

Quality nursing service is a more effective way of inpatient care, and compared with traditional care can be more effective in reducing the length of stay, reduce the incidence of complications, and improve the patient’s satisfaction with the nursing staff.

## Acknowledgments

The authors thank for the support of all the medical staff in the Department of Cardiology of West China Hospital of Sichuan University.

## Author contributions

**Data curation:** Jialin Wang, Zhong Ye.

**Writing – original draft:** Jialin Wang.

**Writing – review & editing:** Qin Xie.
